# Role of Serum Inflammatory Biomarkers in Risk Stratification of Hospitalized Children with Macrolide-Non-Responsive *Mycoplasma pneumoniae* Pneumonia

**DOI:** 10.3390/children13030313

**Published:** 2026-02-24

**Authors:** Jin-Sung Park, Hyo-Bin Kim

**Affiliations:** 1 Department of Pediatrics, Kangwon National University School of Medicine, Kangwon National University Hospital, Chuncheon 24341, Republic of Korea; mokent@naver.com; 2 Department of Pediatrics, Inha University School of Medicine, Inha University Hospital, Incheon 22212, Republic of Korea

**Keywords:** pediatric pneumonia, treatment non-responsiveness, Mycoplasma pneumonia, biomarkers, diagnosis

## Abstract

**Highlights:**

**What are the main findings?**

- Elevated ferritin levels were significantly associated with the increased risk for MNMP.
- When serum inflammatory biomarkers were elevated simultaneously at admission, the risk of developing MNMP was higher.

**What are the implications of the main findings?**

- Identification of hospitalized children with MP who are at risk of macrolide non-responsiveness using inflammatory biomarkers.
- The combined assessment of serum inflammatory biomarkers is more helpful for MNMP risk stratification.

**Abstract:**

Background/Objectives: Macrolide is the first-line treatment in children with Mycoplasma pneumonia; however, macrolide-non-responsive *Mycoplasma pneumoniae* pneumonia (MNMP) has been increasing recently. We aimed to investigate serum inflammatory biomarkers that could identify children at risk of clinically defined macrolide non-responsiveness as early as possible. Methods: This retrospective cohort study included 93 children hospitalized with Mycoplasma pneumonia between September 2019 and January 2020. Patients were classified into macrolide-sensitive MP (MSMP) and MNMP groups based on clinical response to treatment. Clinically defined MNMP was defined as persistent fever and lack of clinical improvement after at least 3 days of macrolide therapy, reflecting macrolide non-responsiveness in routine clinical practice. By reviewing medical records, we compared laboratory findings at admission, including serum procalcitonin (PCT), C-reactive protein (CRP), lactate dehydrogenase (LDH), ferritin, and erythrocyte sedimentation rate (ESR), between the two groups to identify potential predictive biomarkers. Multivariable logistic regression analysis was used to estimate the risk for MNMP based on serum inflammatory biomarkers. Results: CRP, ferritin, and ESR levels at admission were higher in the MNMP group than the MSMP group. By multivariate analysis, elevated ferritin levels were significantly associated with an increased risk of macrolide non-responsiveness. In addition, when serum inflammatory biomarkers were elevated simultaneously at admission, the risk of MNMP was higher. Conclusions: Serum inflammatory biomarkers may assist in early risk stratification of children with clinically defined macrolide non-responsiveness following macrolide therapy. Furthermore, combined assessment of multiple inflammatory biomarkers may improve early risk evaluation.

## 1. Introduction

Mycoplasma pneumonia (MP) is a common pediatric community-acquired pneumonia characterized by outbreaks every 3–5 years in Korea [1,2]. Although most cases display a self-limiting disease course, severe pneumonia with complications requiring careful observation and additional treatment can occur [3]. Macrolides have traditionally been the first-line therapy for MP [4]. The prevalence of macrolide-resistant *M. pneumoniae* pneumonia (MRMP), defined by the presence of resistance-associated 23S rRNA mutations, has increased in East Asia, including Korea [5,6]. In Korea, MRMP was reported to account for 71.3% of hospitalized MP cases in 2019–2020 [5,6]. However, MRMP is a genotypic classification that requires molecular testing, which is not always available or timely in routine clinical practice. Therefore, treatment decisions are often guided by clinically defined macrolide non-responsiveness (MNMP), assessed by persistent fever and/or lack of clinical improvement despite macrolide therapy. Accordingly, in this study, we focused on MNMP as the clinically relevant phenotype for evaluating early predictors of macrolide non-responsiveness in pediatric MP. In East Asia, where macrolide use is higher than in Western countries, the proportion of MRMP is reported to be high, constituting a public health problem [7].

Macrolide-resistant *Mycoplasma pneumoniae* pneumonia (MRMP) is most accurately defined by the presence of 23S rRNA point mutations; however, in routine pediatric practice, resistance is often inferred clinically based on delayed defervescence and symptom persistence despite macrolide therapy. Early identification of children at risk of such non-responsiveness is clinically important, as prolonged inflammation may necessitate alternative therapeutic strategies, including second-line antibiotics or immunomodulatory treatment.

When macrolide treatments for MRMP are ineffective, patients may experience prolonged symptoms, highlighting the need for second-line treatments. In that regard, antibiotics such as tetracycline or fluoroquinolone can be prescribed for MRMP [8,9]; however, given the known adverse effects of tetracycline and fluoroquinolone in children [10,11], their use in pediatric cases is limited. In such cases, where second-line antibiotics are unsuitable or contraindicated, systemic steroids or intravenous immunoglobulin may be considered in an alternative, acting as immunomodulators to inhibit mycoplasma-induced inflammation [12].

To facilitate early treatment decisions for MNMP, easily detectable markers are needed. Although several biomarkers have been studied for early detection of MNMP [13], no definitive success has been achieved. Previous studies have suggested associations between elevated inflammatory biomarkers (e.g., CRP, LDH, ferritin, and pro-inflammatory cytokines) and refractory or severe *Mycoplasma pneumoniae* pneumonia. Nevertheless, findings regarding the prognostic or predictive significance of single biomarkers have been inconsistent, suggesting that no single marker reliably captures early treatment non-responsiveness. Accordingly, we sought to evaluate combinations of clinical and laboratory parameters to identify biomarkers associated with clinically defined macrolide non-responsiveness in the early stage of pediatric MP.

## 2. Materials and Methods

### 2.1. Characteristics of the Participants

We retrospectively analyzed data of children who were diagnosed as having MP and hospitalized in the pediatric department of a single tertiary hospital in Korea from 1 September 2019 to 31 January 2020. Because only hospitalized children were included, milder outpatient cases were excluded, potentially limiting generalizability and enriching the cohort for more severe inflammatory phenotypes.

Data on age, sex, and clinical manifestation of participants were reviewed from medical records. Patients diagnosed with *Mycoplasma pneumoniae* pneumonia were divided into two groups: macrolide-sensitive MP (MSMP) and MNMP. MNMP was defined as cases in which clinical symptoms, particularly fever and other respiratory signs, did not begin to improve after at least 3 days of macrolide therapy. In this study, MNMP was defined clinically based on lack of improvement after macrolide therapy and may reflect a combination of antimicrobial resistance, host immune response, and disease severity rather than confirmed microbiological resistance. In contrast, MSMP was defined as cases that showed clinical improvement, such as resolution of fever and alleviation of symptoms, within 3 days of macrolide treatment. MNMP and MSMP were defined clinically based on treatment response rather than laboratory-confirmed resistance, reflecting real-world pediatric practice where routine molecular testing is not universally available. No molecular testing for 23S rRNA mutations was available in this cohort; therefore, treatment response was used as a pragmatic clinical surrogate in this retrospective analysis.

This study was approved by the Institutional Review Board of Inje University Sanggye Paik Hospital (#SGPAIK2022-07-010), and the requirement for written informed consent was waived due to the retrospective design.

### 2.2. Diagnostic Criteria for Mycoplasma pneumoniae Pneumonia

The diagnosis of MP was confirmed based on respiratory symptoms and lung infiltration visible on chest X-rays and the following positive findings: initial positive serologic immunoglobulin M (IgM) findings or positive conversion from negative IgM mycoplasma antibody, greater than four times increase in the IgG antibody titer from baseline, or positive findings of real-time polymerase chain reaction (RT-PCR) of *Mycoplasma pneumoniae* conducted via nasopharyngeal aspiration or sputum samples [14]. Patients with underlying diseases such as congenital heart disease, chronic lung disease, or immunodeficiency were excluded from the study.

### 2.3. Laboratory Findings

Blood tests included complete blood cell (CBC) with differential counts, serum blood urea nitrogen (BUN), creatinine (Cr), aspartate aminotransferase (AST), alanine aminotransferase (ALT), and bilirubin. In addition, the measurements of blood inflammatory biomarkers such as erythrocyte sedimentation rate (ESR), C-reactive protein (CRP), procalcitonin (PCT), lactate dehydrogenase (LDH), and ferritin were included. The chest X-ray was read by two pediatric doctors who classified the aspects of pneumonia into lobar and bronchial pneumonia and investigated whether there was pleural effusion. All laboratory and radiologic tests used in this study were performed on the first day of admission, before the initiation of macrolide therapy. Radiologic severity, hypoxia, and clinical severity scores were not systematically adjusted for and may independently influence biomarker levels.

### 2.4. Statistical Analysis

Categorical variables between the MSMP and MNMP groups were analyzed using the chi-square test, whereas continuous variables were analyzed using Student’s *t*-test. The cutoff criteria of serum inflammatory markers were determined by plotting a receiver operating characteristics curve, and the odds ratios (ORs) and 95% confidence intervals (CIs) were analyzed using multivariable logistic regression. Significance was set at *p* < 0.05. IBM SPSS Statistics ver. 25.0 (IBM Co., Armonk, NY, USA) was used for the statistical analysis. No formal a priori sample size calculation was performed, and the relatively small sample size may limit statistical power, particularly in multivariable analyses. Given the retrospective design, the study aimed to explore associations rather than to establish definitive predictive models.

## 3. Results

### 3.1. Baseline Characteristics

The characteristics of 93 participants, including 24 and 69 in the MSMP and MNMP groups, respectively, are shown in Table 1. There were 50 male and 43 female participants, and there was no significant difference in the distribution of sexes between the MSMP and MNMP groups. There was no significant intergroup difference in mean age (MSMP, 4.89 ± 3.77 years and MNMP, 6.11 ± 2.75 years, *p* = 0.150), mycoplasma IgM antibody titers, or hematological parameters, such as WBC, BUN, Cr, AST, and total bilirubin, except for neutrophil and platelet counts and ALT, which were not clinically significant. Although some laboratory parameters reached statistical significance, their magnitude was small and unlikely to be clinically meaningful. However, the MNMP group had a higher incidence of pleural effusion than the MSMP group (75.0% vs. 25.0%, *p* = 0.001).

The results of the comparison of serum inflammatory biomarkers, including PCT, CRP, LDH, ferritin, and ESR, between MSMP and MNMP groups, are presented in Table 1. CRP, ferritin, and ESR levels were significantly higher in the MNMP group than in the MSMP group (*p* = 0.049, *p* = 0.014, and *p* = 0.017, respectively). However, PCT and LDH levels did not show significant intergroup differences (*p* = 0.187 and *p* = 0.099, respectively).

### 3.2. Cut-Off Points of Serum Inflammatory Biomarkers

Cut-off levels of serum inflammatory biomarkers were specified depending on the sensitivity and specificity, and each biomarker was divided by the cut-off levels. While the reference values suggested by the manufacturer of each biomarker were 0.5 ng/mL for PCT, 0.3 mg/dL for CRP, 430 U/L for LDH, 300 ng/mL for ferritin, and 9 mm/h for ESR, the newly estimated cut-off levels of serum inflammatory biomarkers were as follows: PCT, 0.101 ng/mL; CRP, 1.95 mg/dL; LDH, 506 U/L; ferritin, 95.38 ng/mL; and ESR, 32.5 mm/h (Table 2).

### 3.3. Association of Individual Serum Inflammatory Biomarkers with Macrolide-Non-Responsive Mycoplasma pneumoniae Pneumonia

The association of each biomarker for MNMP was analyzed and is summarized in Table S1. PCT was not significant in continuous measurement (adjusted OR [aOR] 1.33, 95% confidence interval [CI] 0.91–1.96) but was significant when it was divided by the cut-off level (0.10 ng/mL; aOR 5.60, 95% CI 1.76–17.86). CRP, LDH, and ferritin levels were significantly higher in the MNMP group than in the MSMP group in both continuous and dichotomous groups. In contrast, ESR did not show statistically significant predictability for MNMP in both continuous levels and dichotomous groups (aOR 1.03, 95% CI 0.99–1.06 and aOR 2.62, 95% CI 0.86–8.00, respectively).

The predictability of each serum biomarker for MNMP is summarized in Table 3. PCT, CRP, ferritin, and ESR were significant in univariate analysis (aOR 4.65, 95% CI 1.56–13.90; aOR 3.90, 95% CI 1.37–11.02; aOR 9.93, 95% CI 3.01–32.70; aOR 3.12, 95% CI 1.13–8.56, retrospectively), but only ferritin showed significant result at multivariate analysis (aOR 5.09, 95% CI 1.29–20.03).

### 3.4. Effectiveness of the Predictability by Combining Serum Inflammatory Biomarkers for Macrolide-Non-Responsive Mycoplasma pneumoniae Pneumonia

Each serum inflammatory biomarker was dichotomized according to the cut-off value, and we compared the predictability of the combination of two or more biomarkers for MNMP. The results are summarized in Supporting Information: Table S2, and the representative combinations, including ferritin, are described in Table 4. For clarity, representative biomarker combinations are presented in Table 4, while overlapping combinations have been consolidated in the Supplementary Materials. When ferritin levels exceeded the cut-off value of 95.38, the predictability for MNMP was 9.17. The predictability was 7.70 when ferritin and LDH levels increased together. Moreover, when ferritin, LDH, and CRP levels increased simultaneously, the risk was 9.49. Similarly, when four biomarkers, namely ferritin, LDH, CRP, and PCT levels, were all increased, the predictability was 12.65. When each of the five biomarkers increased, the predictability for MNMP was 6.90. Other biomarkers showed a similar pattern with ferritin, and each biomarker’s result is summarized in Supporting Information: Table S1. The lower odds ratios observed in combinations including multiple biomarkers likely reflect smaller subgroup sizes and wider confidence intervals, rather than reduced biological relevance. These findings should be interpreted cautiously as exploratory results.

**Figure 1:**
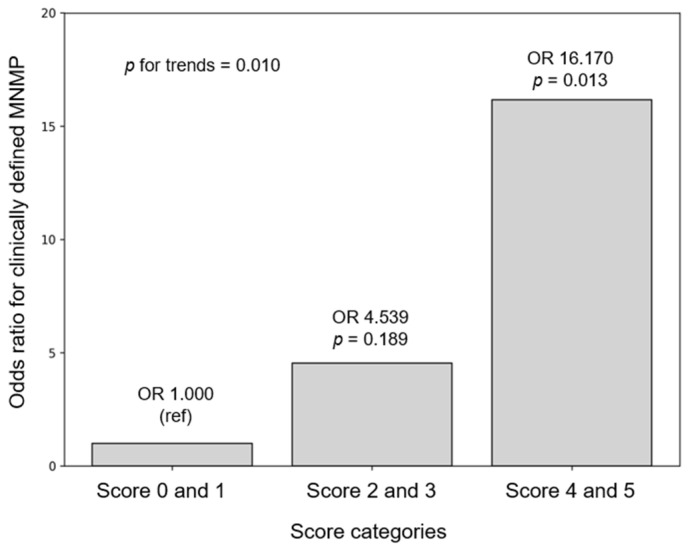
Odds ratios (ORs) for MNMP according to the number of serum inflammatory biomarkers. The score is the number of increased biomarkers according to the cut-off levels.

Furthermore, the risk of MNMP increased to 4.54 when two or three biomarker levels were higher than the cutoff values and to 16.17 when four or five biomarker levels were higher than when none or only one biomarker level was elevated (Figure 1). The biomarker score represents the number of inflammatory biomarkers exceeding predefined cut-off values at admission, reflecting cumulative inflammatory burden rather than pathogen-specific resistance. Figure 1 illustrates the association between the number of elevated biomarkers and the risk of clinically defined MNMP, whereas Table 4 presents odds ratios for specific biomarker combinations. Differences in odds ratios are influenced by subgroup size and model specification. Given the exploratory nature of this study, no adjustment for multiple comparisons or model validation metrics (e.g., AUC, calibration) were applied. Findings should be interpreted cautiously.

## 4. Discussion

In this study, we analyzed serum inflammatory biomarkers that were measured in hospitalized patients for the early detection of MNMP in children. According to the results of our study, when ferritin levels are elevated in Mycoplasma pneumonia, the risk of MNMP is higher. In addition, when various inflammatory biomarker levels increased simultaneously, except for ESR, a stronger association with MNMP was observed. Thus, considering multiple biomarkers simultaneously may improve early risk assessment of MNMP. Rather than proposing novel biomarkers, this study provides incremental evidence that routinely available inflammatory markers, when considered together, may help identify children at risk of early macrolide non-responsiveness in clinical practice.

According to our previous studies comparing the three pandemic periods of Mycoplasma pneumonia, the proportion of MNMP has been increasing, and fever duration and period of improvement after treatment have become longer [15]. Therefore, the number of patients with MNMP who need secondary treatments such as second-line antibiotics (doxycycline or quinolone) or immune-modulating drugs is growing. Notably, identifying predictive factors for MNMP patients requiring secondary treatment as early as possible could prevent the progression of pneumonia by earlier administration of second-line treatment in high-risk cases. Our findings suggest that elevated serum inflammatory biomarkers at admission may be associated with early macrolide non-responsiveness, thereby supporting more individualized and timely treatment decisions.

In a 2018 study in Korea, Jeong and colleagues attempted to identify predictive factors for children who were affected by MNMP, and the authors reported that higher serum PCT and CRP levels correlated with longer fever duration [16]. In another study, LDH, ferritin, and IL-18 levels were higher in the pediatric MNMP group than in the macrolide-sensitive group [17]. We aimed to identify patients with early macrolide non-responsiveness, which may reflect refractory disease rather than microbiologically confirmed resistance with these serum inflammatory biomarkers individually and in combination and analyzed differences by dividing each biomarker into cut-off criteria. In studies from other countries, the independent biomarkers of MNMP were mainly serum CRP, LDH, D-dimer, and peripheral blood neutrophil fractions [18,19]. In addition, increases in serum cytokines IL-6 [20], IL-10 [20,21], IL-17A [22], IL-18 [22,23] and IFN-γ [20,21] were reported to be significant. Studies have reported that lung consolidation or pleural effusion detection in radiological studies [24] or mucus plug formation detected on bronchoscopy might be predictive markers for MNMP. However, these reports only explored each factor as an independent predictor of macrolide responsiveness. A study in China examined the OR of bronchial mucus plug formation when multiple factors were simultaneously satisfied by combining age, duration of fever, CRP, and LDH [25]. The results of our study show that a higher elevation in inflammatory biomarkers, especially in combination, is associated with a greater risk of MNMP.

A meta-analysis investigating the association between host inflammatory biomarkers and the severity of pediatric community-acquired pneumonia suggested that CRP, IL-6, IL-8, and PCT levels were significant biomarkers [26]. However, other meta-analysis study found no significant difference in WBC, CRP, PCT, and LDH levels between MNMP and MSMP in children [27]. In another study, WBC, CRP, and PCT at admission did not differ significantly between Mycoplasma pneumonia and viral pneumonia, but a significant difference was apparent only in comparing PCT segmentally [28]. Although the results of the studies were conflicting, there were significant differences in serum inflammatory biomarkers between the MNMP and MSMP groups in each study. Taken together, previous reports do not seem to provide inflammatory biomarkers that can consistently predict MNMP. Therefore, more research should be conducted in the future. Specifically, we suggest that MNMP is more likely to be predicted if two or more inflammatory biomarkers, including ferritin, are increased. In this study, all patients were treated with clarithromycin as the initial macrolide antibiotic, and macrolide resistance was determined based solely on clinical response. As *Mycoplasma pneumoniae* is difficult to culture and antibiogram testing is not routinely performed in clinical practice, formal susceptibility data were not available. Furthermore, second-line antibiotic susceptibility (e.g., to tetracyclines or fluoroquinolones) was not assessed, as these agents were not used in the initial treatment phase and were beyond the scope of this study. This limitation reflects real-world challenges in managing MNMP and underscores the need for early predictive biomarkers based on initial clinical presentation. Consistent with previous studies reporting higher CRP, LDH, ferritin, and cytokine levels in refractory or MNMP cases, our findings also demonstrate elevated inflammatory markers in clinically non-responsive patients. However, unlike studies relying on molecular resistance testing, our results reflect real-world clinical decision-making based on treatment response. CRP is a non-specific marker of systemic inflammation and may be elevated in various causes of pediatric community-acquired pneumonia. Differences between our findings and previous meta-analyses may be explained by variations in outcome definitions (clinical non-responsiveness vs. molecular resistance), patient populations, timing of biomarker measurement, and disease severity. In this context, CRP may reflect inflammatory burden rather than pathogen-specific resistance. Previous studies have also reported elevated ferritin levels as a biomarker associated with refractory or severe *Mycoplasma pneumoniae* pneumonia, which demonstrated the utility of ferritin in guiding corticosteroid therapy [29,30]. Our findings are consistent with these observations and further extend the evidence by evaluating ferritin within a multi-biomarker framework at hospital admission.

When comparing the manufacturer’s reference values used in hospital laboratories, the sensitivity and specificity of inflammatory biomarkers in predicting disease severity were low. In other studies, LDH was compared with a ≥350 IU/L cut-off value, and the cut-off value for ferritin was set at 230 pg/mL [17]. In our study, the cut-off value was adjusted and compared after considering sensitivity and specificity (Table 2), and the results were clearer than the reference value of the laboratory for predicting macrolide resistance. Thus, appropriate cut-off values of inflammatory biomarkers used to predict MNMP in children should be interpreted as exploratory thresholds that require validation in independent cohorts apart from the manufacturer’s reference levels.

We highlight the quality of our research. While several studies have examined individual biomarkers for predicting MNMP, few have investigated the combined use of multiple serum inflammatory markers [26,27,28]. Our study contributes to this field by demonstrating the potential value of a multi-marker approach for improving early risk assessment. We hypothesized that using a multi-biomarker approach could improve the accuracy of early prediction and better reflect the complexity of the host inflammatory response. Nonetheless, this study had some limitations. First, in our study, macrolide resistance was defined clinically as the absence of improvement after at least 3 days of macrolide treatment, rather than by molecular confirmation of 23s rRNA mutations. While genotypic testing for A2063G mutation is considered the gold standard for detecting macrolide resistance [31], testing gene variation in *Mycoplasma pneumoniae* is not yet clinically commercialized for secondary treatment evaluation. Therefore, creating a tool to determine disease severity based on clinical characteristics and parameters evaluated in initial conventional tests, such as CRP and ferritin, is necessary. Accordingly, our findings should be interpreted as identifying inflammatory patterns associated with clinical non-responsiveness rather than establishing diagnostic criteria for antimicrobial resistance. Because persistent fever was used to define clinical non-responsiveness, and fever is intrinsically associated with systemic inflammatory responses, a degree of circularity cannot be excluded. Therefore, elevated inflammatory biomarkers at admission may reflect an early manifestation of the same inflammatory trajectory rather than serving as independent predictors of treatment failure. Our findings should thus be interpreted as risk stratification markers rather than definitive predictors of resistance. The novelty of this study lies not in identifying new biomarkers, but in examining the combined behavior of routinely available inflammatory markers at admission to stratify the risk of early macrolide non-responsiveness in a real-world clinical setting. Second, the number of participants was small, and this study was conducted retrospectively; therefore, the possibility of preexisting unmeasured bias could not be completely excluded. Given the limited sample size, especially in subgroup analyses, the multivariable models may be subject to overfitting, and odds ratios should be interpreted cautiously. The wide confidence intervals observed further reflect potential instability of the estimates. All analyses were exploratory, and no correction for multiple testing was applied. No formal sample size calculation was performed due to the retrospective nature of the study. Furthermore, the relatively small overall sample size (*n* = 93), especially in subgroup and multivariable analyses, may have reduced statistical power, increased the width of confidence intervals, and contributed to potential model instability. The unequal group sizes may introduce selection bias and limit statistical power, particularly in multivariable analyses. Third, blood samples were collected upon admission; so they were not taken at the same stage of the disease for each individual. Fourth, duration of symptoms prior to admission, viral co-infection, and prior antibiotic exposure were not systematically available and could not be adjusted for; these factors may have influenced biomarker levels.

## 5. Conclusions

This study explored inflammatory markers associated with clinical non-responsiveness in children with MP not controlled by macrolide therapy. Our findings suggest that considering multiple biomarkers simultaneously may assist early risk stratification of children with *Mycoplasma pneumoniae* pneumonia who may show poor clinical response to macrolide therapy to support early clinical decision-making regarding appropriate treatment. Further prospective and large-scale studies related to this topic are needed. These findings should be interpreted within the context of a retrospective single-center cohort without molecular confirmation of resistance.

## Figures and Tables

**Table 1:** Characteristics of participants at baseline.

Total (*n* = 93)	MNMP (*n* = 24)	MSMP (*n* = 69)	*p* Value
Sex			0.963
Male	13 (54.2)	37 (53.6)	
Female	11 (45.8)	32 (46.4)	
Age (years)	6.11 ± 2.75	4.89 ± 3.77	0.150
Hemoglobin (g/dL)	12.13 ± 1.07	12.18 ± 7.77	0.776
WBC (×10^3^/mm^3^)	8620.83 ± 3699.47	9412.17 ± 3681.35	0.367
Neutrophil (%)	65.25 ± 12.42	57.18 ± 14.16	0.015
Lymphocyte (%)	25.47 ± 10.27	31.89 ± 12.95	0.030
Eosinophil (%)	1.39 ± 1.44	1.62 ± 2.14	0.628
Platelet (×10^3^/mm^3^)	267.13 ± 116.62	324.43 ± 98.65	0.022
BUN (mg/dL)	9.68 ± 2.62	9.53 ± 3.10	0.840
Creatinine (mg/dL)	0.38 ± 0.11	0.33 ± 0.10	0.101
AST (U/L)	36.00 ± 13.38	36.80 ± 15.23	0.821
ALT (U/L)	12.33 ± 7.51	17.09 ± 15.01	0.048
Total bilirubin (mg/dL)	0.42 ± 0.17	0.39 ± 0.19	0.452
Mycoplasma IgM	4.35 ± 4.63	3.02 ± 3.42	0.140
Pleural effusion at admission	6 (75.0)	2 (25.0)	0.001
Procalcitonin (ng/mL)	0.64 ± 1.67	0.28 ± 0.91	0.187
CRP (mg/dL)	5.73 ± 7.23	2.58 ± 2.96	0.049
LDH (U/L)	650.13 ± 300.84	542.84 ± 95.60	0.099
Ferritin (ng/mL)	145.6 ± 93.91	90.82 ± 58.92	0.014
ESR (mm/h)	41.38 ± 16.67	30.70 ± 18.89	0.017

Values are presented as *n* (%) or mean ± standard deviation.

**Table 2:** Sensitivity and specificity of serum inflammatory biomarkers according to cut-off levels.

	Reference Level	Cut-Off Level	MSMP, *n* (%)	MNMP, *n* (%)	Sensitivity	Specificity
Procalcitonin (ng/mL)	0.5	0.10	31 (44.9)	19 (79.2)	0.792	0.551
CRP (mg/dL)	0.3	1.95	30 (43.5)	18 (75.0)	0.750	0.565
LDH (U/L)	430	506	36 (52.9)	17 (70.8)	0.708	0.478
Ferritin (ng/mL)	300	95.38	22 (32.4)	19 (82.6)	0.792	0.681
ESR (mm/h)	9	32.5	28 (43.8)	17 (70.8)	0.708	0.594

**Table 3:** Independent factors associated with macrolide-non-responsive *Mycoplasma pneumoniae* pneumonia using logistic regression.

Variables	Univariate		Multivariate	
	OR (95% CI)	*p* Value	OR (95% CI)	*p* Value
Age (per one year)	1.10 (0.96–1.25)	0.151	1.13 (0.90–1.42)	0.993
Male sex (vs. female)	0.97 (0.38–2.48)	0.963	1.00 (0.29–3.42)	0.257
Procalcitonin (ng/mL)	4.65 (1.56–13.90)	0.006	2.74 (0.66–11.35)	0.164
(ref, Low PCT [<0.10])				
CRP (mg/dL)	3.90 (1.37–11.02)	0.010	1.01 (0.22–4.49)	0.988
(ref, Low CRP [<1.95])				
LDH (U/L)	2.15 (0.79–5.87)	0.132	2.29 (0.57–9.17)	0.239
(ref, Low LDH [<506])				
Ferritin (ng/mL)	9.93 (3.01–32.70)	<0.001	5.09 (1.29–20.03)	0.020
(ref, Low ferritin [<95.38])				
ESR (mm/h)	3.12 (1.13–8.56)	0.027	1.58 (0.37–6.67)	0.534
(ref, Low ESR [<32.5])				

Cut-off value of each factor: Procalcitonin, 0.101 ng/mL; CRP, 1.95 mg/dL; LDH, 506 U/L; Ferritin, 95.38 ng/mL; ESR, 32.5 mm/h.

**Table 4:** Predictive value of ferritin for macrolide-non-responsive *Mycoplasma pneumoniae* pneumonia in the participants.

Variables	aOR * (95% CI)	*p* Value
High ferritin group (Ref. Low ferritin group)	9.17 (2.67–31.53)	<0.001
High ferritin and LDH group	7.70 (2.66–22.25)	<0.001
High ferritin, LDH, and CRP group	9.49 (2.95–30.55)	<0.001
High ferritin, LDH, CRP, and PCT group	12.65 (3.52–45.36)	<0.001
High ferritin, LDH, CRP, PCT, and ESR group	6.90 (1.77–26.90)	0.005

* Adjusted for sex and age. Cut-off value of each factor: PCT, 0.10 ng/mL; CRP, 1.95 mg/dL; LDH, 506 U/L; ferritin, 95.38 ng/mL; ESR, 32.5 mm/h.

## Data Availability

The data presented in this study are available on request from the corresponding author. The data are not publicly available due to privacy and ethical reasons.

## Supplementary Materials

The following supporting information can be downloaded at https://www.mdpi.com/article/10.3390/children13030313/s1; Table S1: Predictive factor analysis for the macrolide-non-responsive *Mycoplasma pneumoniae* pneumonia. Table S2: Predictive analysis for macrolide-non-responsive *Mycoplasma pneumoniae* pneumonia by a combination of inflammatory biomarkers.

## Abbreviations

The following abbreviations are used in this manuscript:

MNMP	Macrolide-non-responsive *Mycoplasma pneumoniae* Pneumonia
MRMP	Macrolide-resistant *Mycoplasma pneumoniae* Pneumonia
MSMP	Macrolide-sensitive *Mycoplasma pneumoniae* Pneumonia
WBC	White Blood Cells
BUN	Blood Urea Nitrogen
Cr	Creatinine
AST	Aspartate Aminotransferase
ALT	Alanine Aminotransferase
IgM	Immunoglobulin M
PCT	Procalcitonin
CRP	C-reactive protein
LDH	Lactate dehydrogenase
ESR	Erythrocyte sedimentation rate
