# Supplementary material for: Role of Serum Inflammatory Biomarkers in Risk Stratification of Hospitalized Children with Macrolide-Non-Responsive Mycoplasma pneumoniae Pneumonia
Source: Children (Basel). 2026 Feb 24;13(3):313. doi: 10.3390/children13030313 (PMC13024770; doi:10.3390/children13030313)
Supplement: Supplementary file 1 [file children-13-00313-s001.zip › children-4092624-supplementary.pdf]

Supplementary tables

Table S1. Predictive factor analysis for the MRMP

Variables (as continuous variables)	aOR*	95% CI	*p* value	Variables (as categorical variables)	aOR*	95% CI	*p* value
PCT	1.33	0.91–1.96	0.143	Procalcitonin (ref. < 0.10)	5.60	1.76–17.86	0.004
CRP	1.13	1.02–1.25	0.020	CRP (ref. < 1.95)	3.71	1.25–10.99	0.018
LDH	1.01	1.00–1.01	0.005	LDH (ref. < 506)	5.02	1.39–18.09	0.014
Ferritin	1.01	1.00–1.02	0.014	Ferritin (ref. < 95.38)	9.17	2.67–31.53	<0.001
ESR	1.03	0.99–1.06	0.083	ESR (ref. 32.5)	2.62	0.86–8.00	0.090

*Adjusted for age and sex.

PCT, procalcitonin; CRP, C-reactive protein; LDH, lactate dehydrogenase; ESR, erythrocyte sedimentation rate.

Table S2. Predictive analysis for MRMP by a combination of inflammatory biomarkers

Combination of biomarkers	Participants with all high levels	Others	aOR* (95% CI)	*p* value
PCT + CRP	36	57	4.84 (1.75–13.42)	0.002
PCT + LDH	33	60	7.98 (2.28–27.88)	0.001
PCT + Ferritin	30	63	7.54 (2.63–21.58)	<0.001
PCT + ESR	26	67	4.69 (1.69–13.06)	0.003
CRP + LDH	29	64	5.38 (1.80–16.11)	0.003
CRP + Ferritin	30	63	8.34 (2.66–26.15)	<0.001
CRP + ESR	32	61	3.70 (1.29–10.62)	0.015
LDH + Ferritin	25	68	7.70 (2.67–22.25)	<0.001
LDH + ESR	23	70	5.30 (1.87–15.03)	0.002
Ferritin + ESR	27	66	4.33 (1.46–12.84)	0.008

a) Combination of two biomarkers

*Adjusted for age and sex.

PCT, procalcitonin; CRP, C-reactive protein; LDH, lactate dehydrogenase; ESR, erythrocyte sedimentation rate.

b) Combination of three biomarkers

Combination of biomarkers	Participants with all high levels	Others	aOR* (95% CI)	*p* value
PCT+CRP+LDH	24	69	7.21 (2.27–22.93)	0.001
PCT+CRP+Ferritin	23	70	9.50 (3.01–30.01)	<0.001
PCT+CRP+ESR	23	70	4.79 (1.66–13.85)	0.004
PCT+LDH+Ferritin	20	73	9.49 (2.97–30.31)	<0.001
PCT+LDH+ESR	14	79	8.53 (2.40–30.25)	0.001
PCT+Ferritin+ESR	18	75	4.92 (1.58–15.32)	0.006
CRP+LDH+Ferritin	19	74	9.50 (2.95–30.55)	<0.001
CRP+LDH+ESR	17	76	4.52 (1.47–13.97)	0.009
CRP+Ferritin+ESR	23	70	4.93 (1.63–14.90)	0.005
LDH+Ferritin+ESR	16	77	4.93 (1.56–15.51)	0.006

*Adjusted for age and sex.

PCT, procalcitonin; CRP, C-reactive protein; LDH, lactate dehydrogenase; ESR, erythrocyte sedimentation rate.

c) Combination of four biomarkers

Combination of biomarkers	Participants with all high levels	Others	aOR* (95% CI)	*p* value
PCT+CRP+LDH+Ferritin	16	77	12.65 (3.53–45.36)	<0.001
PCT+CRP+LDH+ESR	13	80	6.82 (1.92–24.25)	0.003
PCT+CRP+Ferritin+ESR	17	76	5.72 (1.78–18.43)	0.003
PCT+LDH+Ferritin+ESR	11	82	6.90 (1.77–26.90)	0.005
CRP+LDH+Ferritin+ESR	14	79	5.04 (1.50–16.89)	0.009

*Adjusted for age and sex.

PCT, procalcitonin; CRP, C-reactive protein; LDH, lactate dehydrogenase; ESR, erythrocyte sedimentation rate.

d) Combination of five biomarkers

Combination of biomarkers	Participants with all high levels	Others	aOR* (95% CI)	*p* value
PCT+CRP+LDH+Ferritin+ESR	11	82	6.90 (1.77–26.90)	0.005

*Adjusted for age and sex
